# Reduced dengue incidence during the COVID-19 movement restrictions in Sri Lanka from March 2020 to April 2021

**DOI:** 10.1186/s12889-022-12726-8

**Published:** 2022-02-24

**Authors:** S. N. Surendran, R. Nagulan, K. Sivabalakrishnan, S. Arthiyan, A. Tharsan, T. T. P. Jayadas, S. Raveendran, T. Kumanan, R. Ramasamy

**Affiliations:** 1 Department of Zoology, Faculty of Science, University of Jaffna, Jaffna, Sri Lanka; 2 Faculty of Applied Science, University of Vavuniya, Vavuniya, Sri Lanka; 3 Department of Geography, Faculty of Arts, University of Jaffna, Jaffna, Sri Lanka; 4 Department of Medicine, Faculty of Medicine, University of Jaffna, Jaffna, Sri Lanka

**Keywords:** *Aedes aegypti*, *Aedes albopictus*, COVID-19 containment measures and dengue incidence, Dengue vectors, Dengue transmission, Jaffna district, Sri Lanka

## Abstract

**Background:**

Dengue is the major mosquito-borne disease in Sri Lanka. After its first detection in January 2020, COVID-19 has become the major health issue in Sri Lanka. The impact of public health measures, notably restrictions on movement of people to curb COVID-19 transmission, on the incidence of dengue during the period March 2020 to April 2021 was investigated.

**Methods:**

The incidence of dengue and COVID-19, rainfall and the public movement restrictions implemented to contain COVID-19 transmission were obtained from Sri Lanka government sources. A Seasonal Autoregressive Integrated Moving Average (SARIMA) model was used to predict the monthly dengue incidence from March 2020 to April 2021 for each of the country’s 25 districts based on five years of pre-pandemic data, and compared with the actual recorded incidence of dengue during this period. Ovitrap collections of *Aedes* larvae were performed in Jaffna city in the Jaffna district from August 2020 to April 2021 and the findings compared with similar collections made in the pre-pandemic period from March 2019 to December 2019.

**Results:**

The recorded numbers of dengue cases for every month from March 2020 to April 2021 in the whole country and for all 25 districts over the same period were lower than the numbers of dengue cases predicted from data for the five years (2015–2019) immediately preceding the COVID-19 pandemic. The number of dengue cases recorded nationwide represented a 74% reduction from the predicted number of dengue cases for the March 2020 to April 2021 period. The numbers of *Aedes* larvae collected from ovitraps per month were reduced by 88.6% with a lower proportion of *Ae. aegypti* than *Ae. albopictus* in Jaffna city from August 2020 until April 2021 compared with March 2019 to December 2019.

**Conclusion:**

Public health measures that restricted movement of people, closed schools, universities and offices to contain COVID-19 transmission unexpectedly led to a significant reduction in the reported numbers of dengue cases in Sri Lanka. This contrasts with findings reported from Singapore. The differences between the two tropical islands have significant implications for the epidemiology of dengue. Reduced access to blood meals and lower vector densities, particularly of *Ae. aegypti*, resulting from the restrictions on movement of people, are suggested to have contributed to the lower dengue incidence in Sri Lanka.

**Supplementary Information:**

The online version contains supplementary material available at 10.1186/s12889-022-12726-8.

## Background

The World Health Organization recently recorded an increasing number of global dengue cases, with 5.2 million reported to it in 2019 [1]. Approximately 70% of dengue cases occur in Asia [1]. *Aedes aegypti* and *Aedes albopictus* are respectively the primary and secondary vectors of dengue and also the vectors of other important arboviral diseases worldwide including chikungunya, yellow fever, Rift Valley fever and Zika [1–3].

**Fig. 1:**
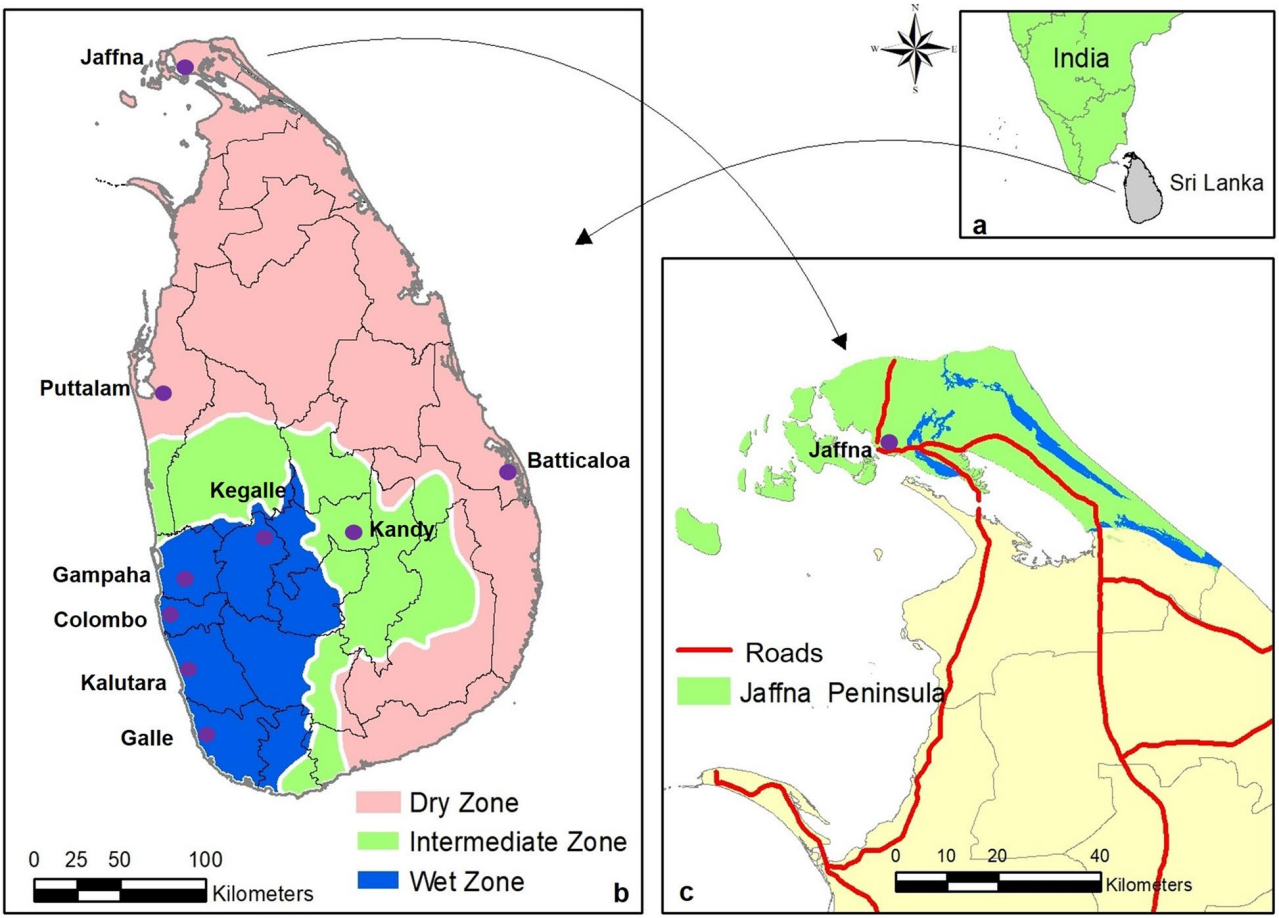
**a** Location of Sri Lanka in the Indian Ocean. **b** The 25 administrative districts and major cities in pertinent districts. The wet, intermediate and dry zones are demarcated. **c** Jaffna peninsula and Jaffna city in North Sri Lanka

Sri Lanka is a dengue-endemic tropical island in the Indian Ocean in proximity to South India and lying between latitudes 5°55′ and 9°51′ N and longitudes 79°41′ and 81°53′ E. It has a population of 21.8 million, a land area of 65,525 km^2^, 25 administrative districts, and is separated by its central hills into dry and wet rainfall zones (Fig. 1). The wet zone, located in the hill country and the Southwest, receives an average annual rainfall of 250 cm in two main rainy seasons, the Northeast monsoon that normally occurs between October and December, and the Southwest monsoon that often begins in April and ends in June. Inter-monsoonal rains also occur between these periods in the wet zone. The dry zone, with an annual rainfall of 60–190 cm, receives most of its rainfall during the Northeast monsoon and typically little or no rain for the rest of the year. An intermediate zone, with mixed characteristics, lies between the dry and wet zones (Fig. 1). The densely populated districts of Colombo, Gampaha and Kalutara are located in the wet zone, while the Jaffna district in the northern Jaffna peninsula lies in the dry zone (Fig. 1). The Jaffna district, with Jaffna as its largest city (population 97,000), includes most of the peninsula and nearby islands, and has a land area of 1100 km^2^ with an average population density of approximately 700 persons/km^2^. Dengue has been present in Sri Lanka from the beginning of the twentieth century and has increased in prevalence throughout the island since the 1990s with all four serotypes (DENV1-4) present and 105,049 cases in 2019 [4]. The Jaffna district recorded 8261 dengue cases in 2019 [4]. The established primary and secondary dengue vectors of dengue in Sri Lanka are also *Aedes aegypti* and *Ae. albopictus* respectively [5–7]. Rainfall is the principal meteorological factor influencing the abundance of dengue vectors throughout Sri Lanka, so that monsoonal rains are followed soon after by a surge in dengue cases [5–7].

Coronavirus disease 2019 (COVID-19) caused by the severe acute respiratory syndrome coronavirus 2 (SARS-CoV-2) was first detected in Wuhan, China in December 2019. COVID-19 has since spread rapidly to become a pandemic that caused approximately 168 million infections and 3.5 million deaths worldwide by 27 May 2021 [8], as well as severe social and economic disruption globally. COVID-19 was reportedly first detected in Sri Lanka on 27 January 2020 [9]. Infections are presently confirmed by PCR tests. Various public health measures to restrict COVID-19 transmission within the country amidst the growing pandemic were taken by the government, and this included advice on improving personal hygiene, social distancing, use of face masks and restrictions on foreigners arriving in the country [9]. Restrictions on movement of people within the country were introduced on 17 March 2020 [9, 10], and these were relaxed or re-imposed with changes in the COVID-19 situation as summarized in Table 1. Vaccination of the public against COVID-19 was begun in a limited manner on 10 February 2021 [9]. A total of 106,484 COVID-19 cases and 667 COVID-19-related deaths were recorded in the country until the end of April 2021 [9].

**Table 1:** Timeline of restrictions on movement of people for suppressing COVID-19 transmission from March 2020 until April 2021 in Sri Lanka

Period	Movement Restrictions	Districts Affected
1–1-2020 to 16–3-2020	None	
17–3-2020 to 20–3-2020	Schools closed nationwide. Stay-at-home^a^ order in Kalutara, Kandy and Puttalam districts	All districts
20–3-2020 to 11–5-2020	Nation-wide stay-at-home^a^ order	All districts
20–4-2020 to 26–5-2020	Prohibition of inter-district travel	All districts
11–5-2020 to 28–6-2020	Night time stay-at-home^a^ order for high-risk districts	Colombo, Gampaha, Kalutara, Kandy, Puttalam and Jaffna
From 11–5-2020	Partial opening of government offices	Colombo, Gampaha, and Kalutara districts
26–5-20 to 28–6-2020	Inter-district public movement allowed except in Colombo and Gampaha districts	Colombo and Gampaha
06–06-2020 to 23–11-2020	Partial opening of schools (grades 5, 11, and 13) except in Colombo, Gampaha and Kalutara districts	Colombo, Gampaha and Kalutara
From 23–11-2020	Partial opening of schools (grades 6 to grade 13) except in Colombo, Gampaha and Kalutara districts	Colombo, Gampaha and Kalutara
29–10-2020 to 9–11-2020	Stay-at-home^a^ order in Colombo, Gampaha and Kalutara districts	Colombo, Gampaha, and Kalutara
12–12-2020 to 18–12-2020	Travel restrictions in selected areas of Galle district	Galle
11–01-2021 to 29–03-2021	All schools opened except in Colombo, Gampaha and Kalutara districts	Colombo, Gampaha and Kalutara
29–03-2021 to 27–04-2021	All schools opened nationwide	
27–04-2021 to 21–10-2021	All schools and educational institutions closed nationwide	All districts

^a^Essential services were allowed to operate and one person from each household was allowed to shop for groceries and medicines during the stay-at-home order. Travel to seek medical advice and treatment was allowed

Dengue is a disease that has to be notified by law to public health authorities of the Ministry of Health in Sri Lanka. Physicians follow the dengue management guidelines developed by the Ministry of Health [11]. An acute onset of fever with two or more of the following clinical manifestations: headache especially retro-orbital pain, myalgia/arthralgia, rash (diffuse, erythematous, macular) and hemorrhage, together with thrombocytopenia are considered sufficient for diagnosing dengue in Sri Lanka [11, 12]. NS1 antigen detection is performed only where possible [11, 12].

The COVID-19 pandemic was expected to divert public health resources from dengue vector control programs, and thereby exacerbate dengue transmission [13]. In this context we analyzed the numbers of officially reported dengue cases in each of the 25 administrative districts of Sri Lanka from March 2020 to April 2021 inclusive, when public health measures restricting population movement to reduce COVID-19 transmission were in place.

## Methods

### Data on dengue and COVID-19 cases, geography and climatic factors in Sri Lanka

Monthly case data for dengue from the January 2015 until April 2021, and COVID-19 from March 2020 until the end of April 2021, were obtained from the Government of Sri Lanka Epidemiology Unit web site [14]. Detailed data for monthly rainfall, temperature and relative humidity for the districts of Colombo, Jaffna and Batticaloa from January 2015 to April 2021 were obtained by request from the Government of Sri Lanka Meteorology Department. Spatial data, district boundaries, roads and lagoons, for the map of Sri Lanka in Fig. 1 were obtained from Sri Lanka Government Survey Department. Spatial data on climate zones of Sri Lanka were obtained from the Meteorology Department by request.

### Prediction of the expected number of monthly dengue cases from March 2020 to April 2021

Even though population movement restrictions to contain COVID-19 transmission were only implemented from 17 March 2020 onward, the entire period from 1 March 2020 to 30 April 2021 was used in our analysis because epidemiological and rainfall data were only available on a whole monthly basis. The Autoregressive Integrated Moving Average (ARIMA) model is widely used for time series data forecasting. An extension of ARIMA that supports the direct modeling of the seasonal component of the series is termed the Seasonal Autoregressive Integrated Moving Average model or SARIMA. The SARIMA model takes overall trends as well as seasonal changes in disease incidence into consideration [15]. Dengue incidence is seasonal and principally associated with rainfall in Sri Lanka [5–7]. Therefore, the anticipated numbers of monthly dengue cases from March 2020 to April 2021 were predicted using the SARIMA model from the recorded dengue case numbers for the corresponding months during the pre-pandemic period from January 2015 to February 2020 in a similar manner to a Malaysian study [15].

SARIMA models (p,d,q) x (P,D,Q) (p is the autoregressive lags, d is the degree of differencing, q is the moving-average lags, P is the seasonal autoregressive lags, D is the seasonal degree of differencing and Q is the seasonal moving-average lags) were established for each district and for the whole country from the monthly reported dengue cases during the pre-pandemic period of 62 months from January 2015 to February 2020. SARIMA modelling and extraction of statistical parameters were performed using Python statistical libraries. A unique SARIMA (1,1,0) (1,1,1) model with the strongest correlation of predicted and actual trends (mean R^2^ > 0.7), was selected from several models with different values of (p,d,q) x (P,D,Q). The constructed SARIMA models were then used to predict the expected number of dengue cases for the 14 months from March 2020 to April 2021, when the movement restrictions shown in Table 1 were in place. This was done for each of the 25 administrative districts as well as the entire country.

### Ovitrap collections of *Aedes* larvae in Jaffna city

Ovitrap-based *Aedes* larval collections were performed in Gurunagar (9°39′12.6″N, 80°01′03.5″E), a coastal municipal ward in a built-up area of Jaffna city from August 2020 to April 2021, essentially as previously done in Gurunagar in 2019 [7]. Conventional black plastic ovitraps (capacity: 650 ml, radius: 4.5 cm, height: 10 cm) containing 300 ml of water obtained from the nearest domestic water supply (i.e., well or tap) with a 2 × 15-cm plywood paddle resting against the inside upper rim were placed as described [7]. Ten ovitraps were placed and fortnightly larval collections were made from each ovitrap. Collected *Aedes* larvae were brought to the laboratory of the Department of Zoology, University of Jaffna. Larvae were maintained here under contained insectary conditions, and emerging adult mosquitoes identified at the species level with a standard key as previously described [7].

### Statistical analyses

The non-parametric Wilcoxon signed-rank test was used to determine significant differences between predicted and actual dengue cases considering data for each month from March 2020 to April 2021 for the whole island and also for each of the 25 districts. *Aedes* larval collection data from the period of COVID-19 movement restrictions from August 2020 to April 2021 were compared with corresponding published data for the pre-pandemic period from March 2019 to December 2019 [7]. Because of the small total numbers involved, the Fisher’s exact test was used to determine significance of the differences in the proportions of positive ovitraps during the two periods separately for *Ae. aegypti* and *Ae. albopictus.* The non-parametric Mann–Whitney U test was used to compare the numbers of *Ae. aegypti* and *Ae. albopictus* collected from ovitraps during the pre-pandemic period of March 2019 to December 2019 with the pandemic period of March 2020 to April 2021. All analyses were performed using R statistical software (V4.0.1).

## Results

### Dengue incidence during the pre-pandemic period and from March 2020 to April 2021 with pandemic-related movement restrictions

**Fig. 2:**
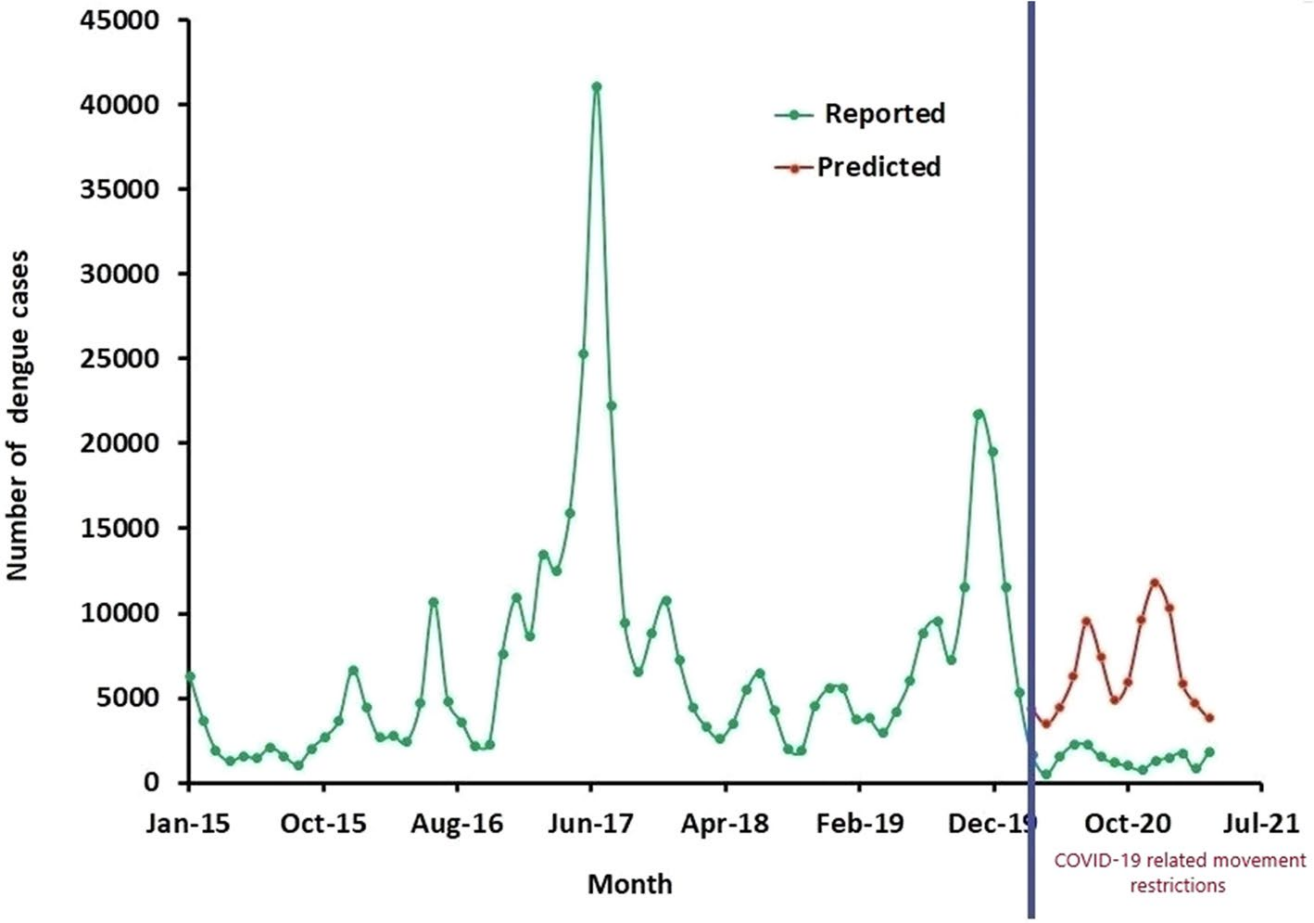
Reported and predicted numbers of monthly dengue cases for the whole of Sri Lanka

The monthly predicted and reported numbers of dengue cases from January 2015 to April 2021 for the whole country are illustrated in Fig. 2 with details provided in Table S1. Peaks of dengue incidence follow soon after the two monsoons and the number of cases is also influenced by the location of the populous districts of Colombo, Gampaha and Kalutara in western Sri Lanka (identified by their principal cities in Fig. 1b) that are subject to both the Southwest and Northeast monsoons [6]. The numbers of reported dengue cases and the numbers predicted from the SARIMA model for the period of COVID-19 movement restrictions from March 2020 to April 2021 are presented in detail in Table S1.

The results show that the actual numbers of dengue cases reported in the whole country during the 14 months of movement restrictions was 21,094 whereas the SARIMA model predicted 82,169 cases for this period, and this represented a 74% reduction during the COVID-19 related movement restriction period (Table S1). The Wilcoxon signed-rank test revealed that the predicted number of dengue cases was significantly higher than the actual number of dengue cases reported in the whole country during the 14-month period (*P* = 0.00012).

### Dengue cases in each district during the COVID-19 movement restrictions

**Fig. 3:**
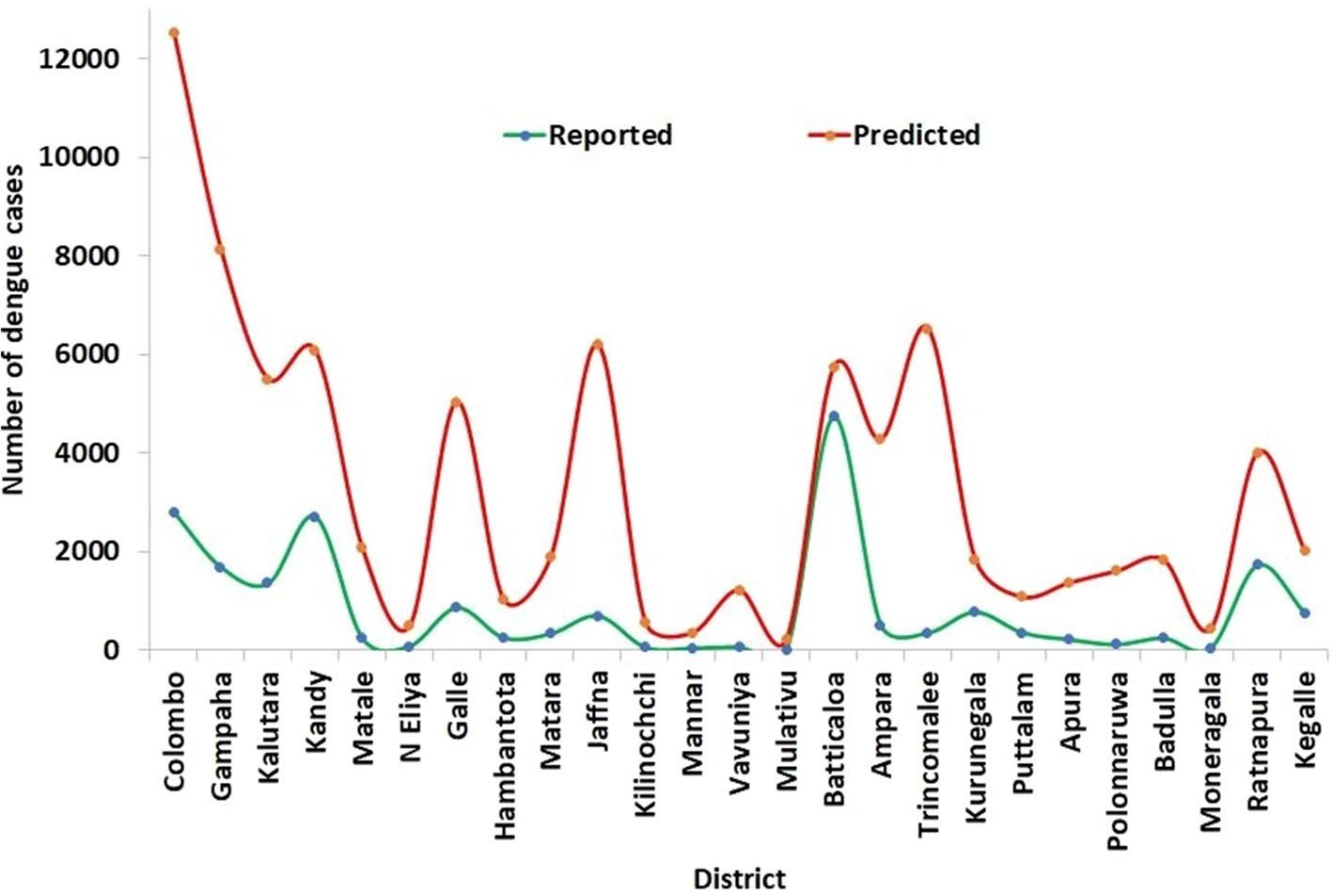
Reported and predicted numbers of dengue cases for each district from March 2020 to April 2021

The total numbers of predicted and reported numbers of dengue cases during the 14-month period of COVID-19 movement restrictions for each of the 25 districts is illustrated graphically in Fig. 3 with detailed numbers provided in Table S2.

The detailed results in Table S2 showed that there was a reduction ranging from 55 to 95% in the reported numbers of dengue cases in different districts with the exception of Batticaloa district where the reduction was 18%. The predicted and actual dengue cases for every month during March 2020 to April 2021 for each of the 25 districts are shown in Table S3. The Wilcoxon signed-rank test considering predicted and actual dengue cases of each month during the 14-month period for each district showed that the actual number of reported dengue cases were significantly lower than the predicted number of dengue cases for all districts (*P* range from < 0.0001 to 0.0085, Table S3), except for Batticaloa (*P* = 0.326, Table S3).

### Dengue incidence in relation to rainfall and COVID-19 movement restrictions in the Jaffna district

**Fig. 4:**
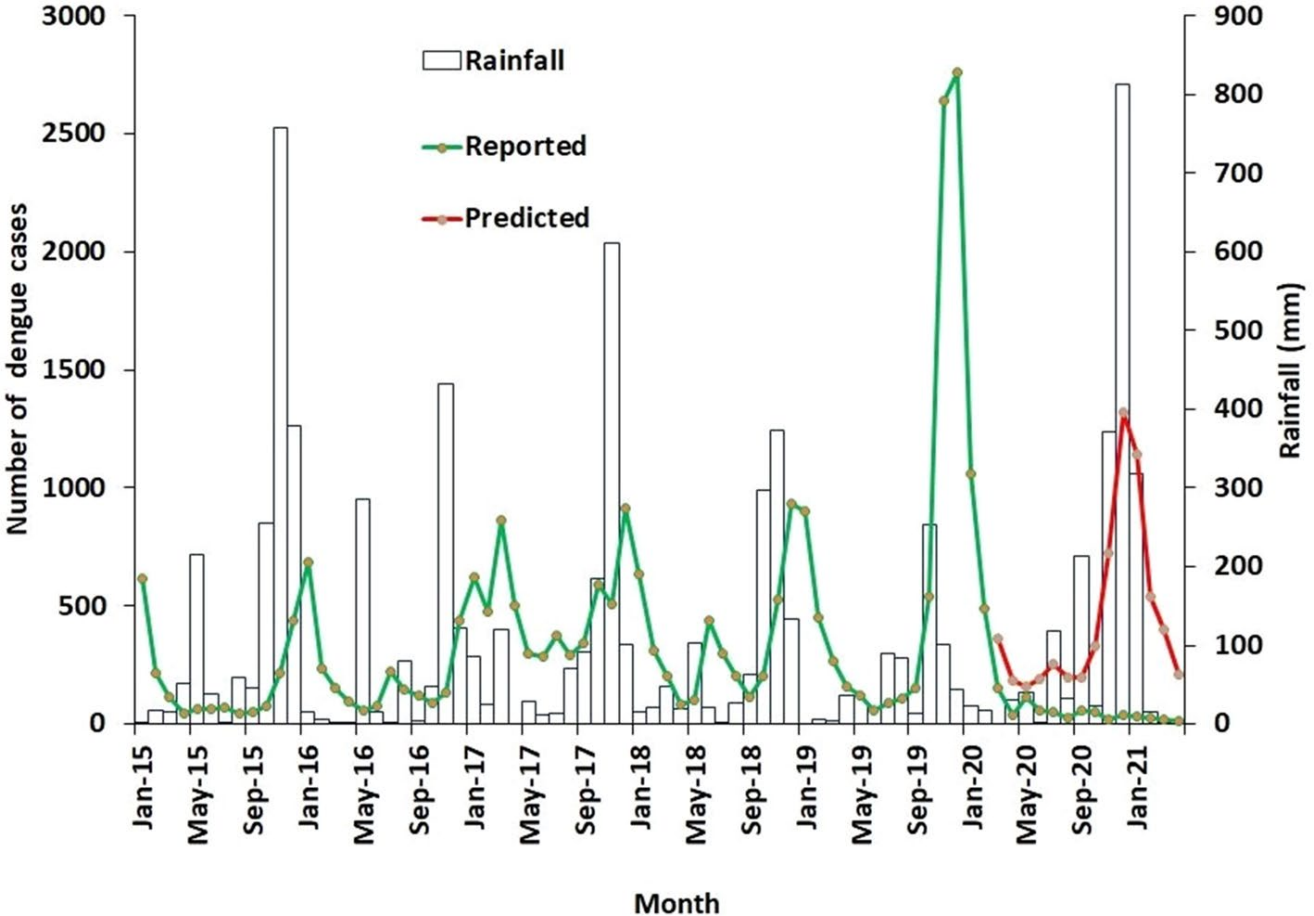
Reported and predicted numbers of monthly dengue cases and monthly rainfall in the Jaffna district

Figure 4 shows the monthly numbers of reported and predicted dengue cases and the monthly average rainfall in the Jaffna district during the five pre-pandemic years and the period of COVID-19-related movement restrictions. Dengue incidence in the Jaffna district is seasonal and typically increases soon after the Northeast monsoon which prevails from October to December [6, 7]. The rainfall pattern in 2020 was similar to that in the pre-pandemic period of 2015 to 2019 in the Jaffna district, but the number of dengue cases reported in the Jaffna district was significantly reduced by 89% in the period of COVID-19-related movement restrictions from March 2020 to April 2021 when compared with predictions based on the five previous pre-pandemic years (Table S2 and Table S3).

### Variations in temperature and relative humidity in Colombo, Jaffna and Batticaloa districts

The Colombo district in the wet zone (Fig. 1) typically exhibits a high dengue incidence. During the 2015–2019 pre-pandemic five year period and the March 2020 to April 2021 pandemic period, it recorded average monthly temperatures of 30.7 °C (range 30.0 – 33.3 °C) and 31.5 °C (range 30.0 – 33.3 °C) and average monthly relative humidities of 80.4% (range 72—86%) and 78.9% (range 71—84%) respectively, while the Jaffna district in the dry zone (Fig. 1), recorded average monthly temperatures of 31.7 °C (range 28.4—35.7 °C) and 31.5 °C (range 28 – 34.4 °C) and average monthly relative humidities of 73.7% (range 64—85%) and 73.6% (range 66—81%) respectively.

The Batticaloa district in the dry zone (Fig. 1) recorded average monthly temperatures of 31.8 °C (range 28.2—35.1 °C) and 31.6 °C (range 28.7 – 34.0 °C) and average monthly relative humidities of 70.5% (range 69—85%) and 74.6% (range 65—85%) during the 2015–2019 pre-pandemic five-year period and the March 2020 to April 2021 pandemic period, respectively.

### *Aedes* larval collections from ovitraps during the August 2020 to April 2021 period in Jaffna city

In the period August 2020 to April 2021, a total of 90 and 282 *Ae. aegypti* and *Ae. albopictus* were respectively collected from the ten ovitraps in Gurunagar in Jaffna city. A similar ovitrap collection from March 2019 to December 2019 in Gurunagar recorded 2380 and 1320 *Ae. aegypti* and *Ae. albopictus* respectively in nine ovitraps (details in Table S4). Both collections covered the Northeast monsoon period from October to December which is the main rainy season in the Jaffna district. However, it was only possible to begin the 2020 collection in August 2020, which was approximately 15 weeks after the initial introduction of COVID-19-related movement restrictions in the country. The comparison of *Aedes* larval collection data from March 2019 to December 2019 in the pre-pandemic area with August 2020 to April 2021 during the pandemic (Table S4) showed (i) an 88.6% reduction in the average monthly sum of the larvae of both *Aedes* species collected per ovitrap during the COVID-19-related movement restrictions (ii) significantly lower proportion of ovitraps with *Ae. aegypti* and *Ae. albopictus* larvae (Fisher’s exact test *P* < 0.0001) during the COVID-19 restrictions, and (iii) collection of significantly reduced numbers of *Ae. aegypti* per ovitrap in comparison to *Ae. albopictus* per ovitrap (Mann–Whitney U test, *P* = 0.032) during the COVID-19 movement restrictions, whereas, previous data [7] (Table S4) showed that significantly greater numbers of *Ae. aegypti* than *Ae. albopictus* per ovitrap were collected at the same location during the pre-pandemic 2019 period (Mann–Whitney U test, *P* = 0.0008).

## Discussion

Numerous non-pharmaceutical public health measures were introduced early in the pandemic in almost all countries to suppress the spread of COVID-19. Various public health measures were also imposed in Sri Lanka, to control the rise in the number of COVID-19 cases in March 2020 [9]. These were supplemented with vaccination that became possible only towards the end of 2020 in Sri Lanka [9]. The urgent need to control COVID-19 diverts resources away from other health sector activities, including the programs to control vector-borne diseases. One documented impact has been the significant increase in the incidence and mortality from malaria during the COVID-19 pandemic period in Zimbabwe [16]. Public health measures applied to reduce COVID-19 transmission can be expected, on the other hand, to decrease the incidence of other infectious diseases transmitted from person to person. This is borne out by marked reductions in the incidence of influenza in diverse countries in both the northern and southern hemispheres [17–19].

Reports on the impact of COVID-19 containment measures on the incidence of dengue have been variable, with increases in dengue incidence observed in Thailand and Singapore [20, 21] and a decrease in Malaysia [15]. Data for the second quarter of 2020 suggested that the incidence of dengue decreased in Sri Lanka during that period of the COVID-19 pandemic [22]. Our results for the period of COVID-19 related movement restrictions from March 2020 to April 2021 nationwide and district-wise, show a significant reduction in the reported number of dengue cases compared with predictions based on the preceding five pre-pandemic years from 2015 to 2019.

There is epidemiological evidence to suggest that movement of people outside of their homes is one of the factors that drive the transmission of dengue virus [23], which is also supported by mathematical modeling studies [24]. Dengue vectors are highly prevalent in premises of schools, hospitals, government offices, transport hubs and factories in Sri Lanka [5, 7]. The anthropophagic *Ae. aegypti* and the partly anthropophagic *Ae. albopictus* exhibit two peaks of blood feeding activity in the morning and afternoon [25], that coincide with schooling and working hours. Our findings in the Jaffna district and elsewhere in Sri Lanka are consistent with restrictions on movement of people leading to a reduction in dengue transmission [23, 24]. There is no reason to expect that other public health measures like the use of face masks, thorough hand washing and social distancing will have had an impact on the transmission of dengue by *Aedes* vectors. However, other possible interacting factors need also to be considered: (i) Early COVID-19 and dengue manifest common clinical symptoms posing a problem for differential diagnosis [26–28]. It is therefore possible that the decrease in reported number of dengue cases in 2020 and 2021 during the COVID-19 pandemic in Sri Lanka can be due in part to the reluctance of people with mild dengue to seek medical treatment for fear of being identified as COVID-19 patients and quarantined; (ii) Dengue transmission in Sri Lanka is mainly controlled through the elimination or minimization of larval development habitats and the application of larvicides by public health staff. However, these efforts were hampered by the COVID-19 containment measures in the Jaffna district and the rest of Sri Lanka and therefore could not have contributed to reducing the incidence of dengue from March 2020 to April 2021; (iii) Reduced access to medical treatment (and therefore testing and recording of dengue) due to the various COVID-19-related movement restrictions. Good public healthcare facilities are readily accessible in most rural and urban areas of Sri Lanka, and its population has been long-accustomed to rapidly seeking medical help for mosquito-borne diseases like malaria [29, 30] and dengue [12]. While the public were permitted to seek medical advice and care during the COVID-19 movement restrictions, access to medical clinics was probably made more difficult by the lack of public transport. This could have contributed to an extent to the reduction in the number of reported dengue cases; (iv) Restrictions on the normal movement of people outside of homes can result in reduced access of vectors to human blood meals as *Ae. aegypti* and *Ae. albopictus* are both exophilic and *Ae. aegypti* is almost exclusively anthropophagic [31]. Diminished blood feeding will reduce oviposition and vector densities, particularly in the case of anthropophagic *Ae. aegypti.* This is consistent with the ovitrap collection data from Gurunagar, Jaffna city, notably the 88.6% fall in *Aedes* larval collection during the period of movement restrictions. Mathematical modelling shows that significantly lower *Aedes* densities and their reduced access to blood meals can markedly lower DENV transmission rates [32]. We propose this as the principal explanation for our findings, but more extensive vector data from different locations in the country are needed to firmly establish this.

Sri Lanka is a tropical island lying between the latitudes 5^o^51’N and 9^o^51’N. There is little variation in monthly temperature and relative humidity except for small falls in temperature and small increases in relative humidity during the monsoon season, as exemplified by data for the Colombo, Jaffna and Batticaloa districts analysed here. Pronounced temperature and relative humidity variations during the year that can influence the transmission of mosquito-borne diseases in other climates [32], do not therefore have a significant role in Sri Lanka. Rainfall is the principal meteorological factor governing dengue incidence in Sri Lanka as shown by previous studies [6, 7, 33] and the present findings. The rainfall pattern in the immediate pre-pandemic five years in most of Sri Lanka as well as the Jaffna district was not markedly different from that in pandemic period of March 2020 to April 2021. This does not support a role for rainfall variation in causing the reduced incidence of dengue throughout Sri Lanka from March 2020 to April 2021. However, exceptionally heavy rainfall in the Batticaloa district during the Northeast monsoon in 2020-2021, was associated with higher than usual numbers of dengue cases [34], which is reflected in our present findings.

Our findings from Sri Lanka are in contrast with data showing that dengue transmission increased during COVID-19 movement restrictions in Singapore [21]. Singapore, like Sri Lanka, is a tropical island lying near the equator and with similar meteorological variations in a year. However, Singapore is a small city state with many offices located in high rise buildings and robust measures in place to limit *Aedes* vector populations, so that public transport and work places are kept relatively free of *Aedes*. Available data suggest that dengue transmission mainly occurs in or around residences in Singapore, where many people were confined during the COVID-19-related movement restrictions [21]. In contrast, Sri Lanka has a large rural area, a considerable rural working population, longer commuting distances for workers in large cities like Colombo and less effective vector control measures in many parts of the country, including the Jaffna district [7]. Movement restrictions to contain COVID-19 transmission in Singapore were also not identical to those applied in Sri Lanka. These factors may be responsible for the different impacts of COVID-19 movement restrictions on the incidence of dengue between Singapore and Sri Lanka.

## Conclusion

During the March 2020 to April 2021 period of COVID-19-related movement restrictions, a reduction in the numbers of reported dengue cases, in comparison with predicted numbers from the preceding five-year pre-pandemic period was seen in all 25 districts of Sri Lanka. This decrease was statistically significant in 24 of the 25 districts. It is proposed that restrictions on the movement of people outside homes which resulted in reduced blood feeding and densities of *Aedes* vectors was important for lowering dengue incidence.

## Supplementary Information

**Additional file 1:** **Supplemenatry Table S1.** Monthly predicted and actual numbers of dengue cases in Sri Lanka during the period March 2020 to April 2021.

**Additional file 2:** **Supplementary Table S2.** Actual and predicted numbers of dengue cases in each of the 25 districts from March 2020 to April 2021.

**Additional file 3:** **Supplementary Table S3.** Monthly predicted and actual number of dengue cases in each district from March 2020 to April 2021.

**Additional file 4:** **Supplementary Table S4. ***Aedes aegypti* and *Ae. albopictus* larval collections from ovitraps in Gurunagar in March 2019 to December 2019 and from August 2020 to April 2021.

## Data Availability

All data generated during this study are included in this published article and its supplementary files.

## Abbreviations

COVID-19: Coronavirus disease 2019
DENV: Dengue virus
NS1: Non-structural protein 1
SARS-CoV-2: Severe acute respiratory syndrome corona virus 2
