# Supplementary material for: Reduced dengue incidence during the COVID-19 movement restrictions in Sri Lanka from March 2020 to April 2021
Source: BMC Public Health. 2022 Feb 24;22:388. doi: 10.1186/s12889-022-12726-8 (PMC8866919; doi:10.1186/s12889-022-12726-8)
Supplement: Supplementary file 4 — Additional file 4: Supplementary Table S4. Aedes aegypti and Ae. albopictus larval collections from ovitraps in Gurunagar in March 2019 to December 2019 and from August 2020 to April 2021. [file 12889_2022_12726_MOESM4_ESM.docx]

**Supplementary Table S4:** *Aedes aegypti* and *Ae. albopictus* larval collections from ovitraps in Gurunagar in March 2019 to December 2019 and from August 2020 to April 2021.

| **Month** | **2019*** | | | | **2020** | | | | **2021** | | | |
| --- | --- | --- | --- | --- | --- | --- | --- | --- | --- | --- | --- | --- |
|  | ***Ae. aegypti*** | | ***Ae. albopictus*** | | ***Ae. aegypti*** | | ***Ae. albopictus*** | | ***Ae. aegypti*** | | ***Ae. albopictus*** | |
|  | **No of larvae** | **+ve** | **No of larvae** | **+ve** | **No of larvae** | **+ve** | **No of larvae** | **+ve** | **No of larvae** | **+ve** | **No of larvae** | **+ve** |
| January | nd |  | nd |  | nd |  | nd |  | 0 | 0/10 | 70 | 4/10 |
| February | nd |  | nd |  | nd |  | nd |  | 0 | 0/10 | 6 | 1/10 |
| March | 109 | 6/9 | 7 | 1/9 | nd |  | nd |  | 0 | 0/10 | 16 | 1/10 |
| April | 289 | 9/9 | 117 | 4/9 | nd |  | nd |  | 0 | 0/10 | 58 | 3/10 |
| May | 285 | 9/9 | 132 | 7/9 | nd |  | nd |  |  | |  |  |
| June | 246 | 7/9 | 109 | 6/9 | nd |  | nd |  |  |  |  |  |
| July | 235 | 9/9 | 170 | 6/9 | nd |  | nd |  |  |  |  |  |
| August | 261 | 8/9 | 160 | 5/9 | 22 | 2/10 | 18 | 2/10 |  |  |  |  |
| September | 274 | 9/9 | 205 | 6/9 | 56 | 3/10 | 40 | 2/10 |  |  |  |  |
| October | 281 | 9/9 | 181 | 6/9 | 12 | 1/10 | 0 | 0/10 |  |  |  |  |
| November | 199 | 8/9 | 184 | 5/9 | 0 | 0/10 | 56 | 3/10 |  |  |  |  |
| December | 201 | 6/9 | 55 | 5/9 | 0 | 0/10 | 18 | 1/10 |  |  |  |  |
| **Total** | **2380** |  | **1320** |  | **90** |  | **132** |  | **0** |  | **150** |  |

nd**–** not done; **+ve** - positive ovitraps out of number of ovitraps placed;

* - 2019 data are from Reference 7. Surendran et al. 2021.
